# Long-Term Influences of Stunting, Being Underweight, and Thinness on the Academic Performance of Primary School Girls: The NW-CHILD Study

**DOI:** 10.3390/ijerph18178973

**Published:** 2021-08-26

**Authors:** Xonné Haywood, Anita Elizabeth Pienaar

**Affiliations:** 1 Physical Activity, Sport and Recreation Focus Area, Faculty of Health Science, North-West University, Potchefstroom 2520, South Africa; hxonne@yahoo.com; 2 School of Human Movement Sciences, North-West University, Potchefstroom 2520, South Africa

**Keywords:** academic achievement, primary school girls, undernutrition, stunting, wasting, thinness

## Abstract

Poor socio-economic status contributes to undernutrition which, in turn, can increase the risk of academic underachievement. This study wants to determine if stunting, being underweight, and thinness show long term relations with academic performance in primary school girls aged 6 to 13 in the North West province of South Africa. A randomized and stratified longitudinal research design including a baseline and two time-point measurements over seven school years was used. The sample included girls aged 6 to 13 years (*N* = 198) in the North West province of South Africa. Academic performance in the June school assessments and national and provincial assessments in grades 1, 4, and 7 were used to determine academic performance. Independent *t*-testing was used to determine differences between thinness, underweight and stunted girls as opposed to a reference group with no undernutrition indices. A repeated measures ANOVA and post hoc Bonferroni adjustment analyzed relations over time. Normal weight girls significantly outperformed stunted girls academically (*p* < 0.05) over time. Stunting had prolonged and significant negative influences on language, mathematics, and grade point average (*p* < 0.05). Early identification of undernutrition, especially stunting, is important for intervention and the implementation of timely prevention strategies, especially during early childhood years.

## 1. Introduction

Nutritional deficiencies during early childhood are associated with poor brain development [1,2,3]. Undernutrition is characterized by stunting and thinness, where stunting is described as being short in height relative to age [2,3]. Being underweight is defined in terms of grades of thinness (grades 1–3) and is characterized by rapid weight loss or an inability to increase body mass [4]. Both these conditions are associated with poor academic performance in mathematics, reading, and writing skills [5]. In addition, sick or undernourished children regularly display poor school attendance or struggle with motor functioning skills, concentration, problem solving skills, and recall during tuition, adding to scholastic underperformance [1,5]. Poor academic performance during childhood has rippling effects with long-term consequences, inter alia, acquiring poor educational qualifications, which could have a detrimental effect on psychosocial development in adulthood [6].

Several studies that specifically investigated the relation between academic performance and undernutrition are based on cross-sectional designs [5,7,8]. South African cross-sectional studies confirmed negative influences of undernutrition and thinness on the academic performance of boys and girls [5,8]. Pienaar [5] recorded a positive relation between stunting (HAZ: height-for-age z-score) and mathematical performance of grade 1 learners in the North West province that included both high and low socio-economic participants, although no gender specific relationships were reported.

A few studies also investigated long term influences of undernutrition on academic performance and found that these conditions can develop into chronic disorders, hamper cognitive development, or even lead to premature death over the long term [9,10,11,12]. Poor socio-economic status (SES) is furthermore confirmed as a contributing factor to undernutrition, which again gives rise to poorer academic performance [13,14].

Statistics from the World Health Organization (WHO) show that undernutrition is a worldwide phenomenon [15]. Africa is, however, the only continent where stunting increased from 2000 to 2016, with supporting statistics confirming an increase from 50.4 million to 59 million [2].

A systematic review in Sub-Saharan Africa confirmed in this regard the existence of malnutrition and being underweight among 5 to 17-year-old-children in Africa, of which 20% live in the Sub-Saharan region [16]. Results showed that Africa displays limited improvement (17% decrease) in stunting in comparison with other continents such as Asia that reported decreases of 36%, LAC (Latin American Countries), and the Caribbean countries (39%) [2].

In South Africa, the SANHANNES-11 [17] survey (The South African National Health and Nutrition Examination Survey) reported stunting in 19.2% of children between the ages of 0 to 14 years. The more recent 2016 HAKSA report (Healthy Active Kids South Africa) confirms malnutrition as a remaining problem [18] where one in five children are reported to still suffer from stunting. This report showed that the prevalence of undernutrition remained relatively stable from 2014 to 2106, but remains high, especially among children from low socio-economic status (SES) and boys living in rural areas [18]. A recent systematic literature review reveals that women are particularly vulnerable to food insecurity [19]. South African statistics (2016) show that women between the ages of 25–64 represent the largest portion of the population without any childhood and adolescence tuition while more women than men are also illiterate. One possible explanation for this trend is undernutrition [20]. Malnutrition in the form of undernutrition and hidden hunger is again simultaneously the direct outcome and consequence of poverty [21].

A high prevalence of undernutrition is therefore a reality worldwide but also in South Africa [2,15,16,17,18,22,23,24], which is one of the countries that is most seriously affected by these conditions [2,18]. However, limited resources make it difficult to address undernutrition and thinness in South Africa with similar intervention programs to those in developed countries [25].

The burden of undernutrition on the future of developing children via the relation between undernutrition and academic performance are also confirmed, although mostly through cross-sectional studies [8,13,26]. Evidence about the relation between undernutrition and academic performance among girls aged 7 to 13 is, however, very limited [5,7,13].

This study aimed to determine the longitudinal relationship between academic performance and different indices of undernutrition over the primary school period, specifically in girls between 6 and 13 years in the North West province of South Africa. Only a few studies have been published worldwide with similar objectives but with a focus on different age groups and SES relationships, while no gender specific analyses were done [9,10,23,27]. In addition, the TIMSS report (Trends in International Mathematics and Science Society) [28] also still reveals contradictions regarding relationships between mathematic performance, gender, and SES in different countries.

## 2. Materials and Method

### 2.1. Investigating Group and Procedures

**Figure 1:**
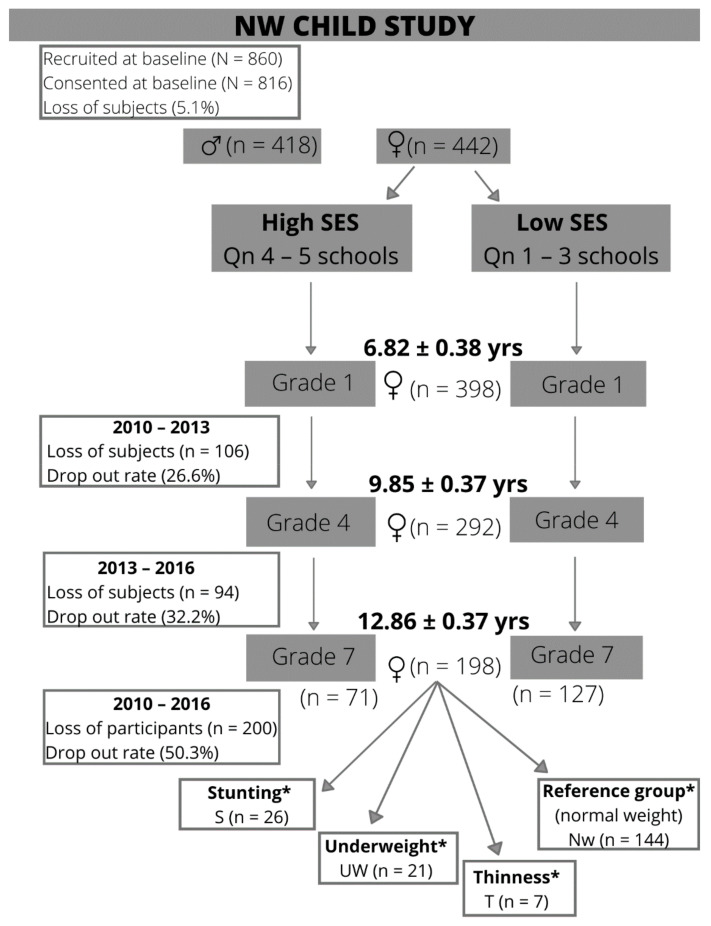
Flow diagram for participant recruitment of the NW-CHILD study, 2010–2016. * Participants with malnutrition over seven years. Excludes participants that moved into the normal weight category over the seven-year period.

The study is based on a longitudinal research design which forms part of the NW-CHILD study (North West Child Health Integrated with Learning and Development), which represents the primary school period of seven school years. Stratified and random sampling was used to determine the study population. Stratification of the sample was based on school district, school quintile (1–5), and gender. The Department of Basic Education (DBE) supplied a list of all the schools in the North West Province (NWP) for the sample to be compiled. The NWP is divided into eight school districts which, in turn, include 12 to 22 sub districts, respectively, with approximately 20 schools per district (minimum 12 and maximum 47). South Africa also makes use of a poverty classification system to categorize the schools into an appropriate quintile status based on SES factors. The schools’ quintile status is aligned with the National Poverty Table sourced from the National Census data, which includes income, dependency ratios, and literacy levels. This poverty classification is used by the DBE in all the provinces to classify schools into various quintiles. Schools are categorized into five quintiles, with quintile 1 schools representing schools with a low SES and quintile 5 representing schools with a high SES. School quintiles 1 and 2 represent the poorest schools that are exempted from school fees and that are supported by feeding schemes on school days [29]. This study used the quintile school status to categorize the participants’ SES. Only the participants who had full data sets that included baseline and the two follow-up time points were included for the purposes of this study. The baseline measurements were done in 2010 in the grade 1 year of the participants, with the first time point measurements in 2013 representing the group’s grade 4 year, and the second time point measurements in 2016 at the end of the primary school year in grade 7. Figure 1 represents a flow diagram that shows the recruitment process, and the loss to follow-up over the seven year school period to arrive at the final group of participants over the follow-up period that was categorized into different categories of undernutrition.

Eight hundred and sixty children were recruited for participation in the study in 2010, of which 816 (418 boys and 398 girls) with a mean age of 6.82 (SD = 0.38) took part. The parents of 829 learners consented to participation, but only 816 (dropout rate of 5.1%) took part due to absence on the day of testing or exclusion because of incorrect ages.

At time point 1 in 2013, 574 participants consisting of boys (n = 282 of 49.12%) and girls (n = 292 of 50.87%) with a mean age of 9.85 (SD = 0.38) took part, representing a dropout rate of 29.9% over three years. At time point 2 in 2016 a further drop out of 28.1% occurred (n = 229). The total group dropout rate from 2010 to 2016 was 53.31% (n = 435).

This study only focused on the 198 girls who participated in all three measurements, representing a dropout rate of 50.3% (n = 200) over the seven year school period. Low SES schools (quintile 1 = 51), (quantile 2 = 28), and (quintile 3 = 48) represented 63.82% of the girls (n = 127), while 35.68% (n = 71) represented high SES schools (quintile 4 = 32 and quintile 5 = 39) in this study. The participants had a mean age of 6.82 (SD = 0.38) years in grade 1, 9.85 (SD = 0.37) years in grade 4 and 12.86 (SD = 0.37) in grade 7.

### 2.2. Ethical Considerations

Ethical approval was granted by the NWU-Health Research Ethics Committee of the Faculty of Health Sciences of the North-West University (00070-09-A1) as well as the Department of Basic Education of the North West province of South Africa. School principals granted permission and continued permission for the follow-up measurements. Every parent/ guardian and child had to provide consent and assent as well as re-consent for participation in the study. The participants also had an opportunity to ask questions and were informed that they could leave the study at any time.

### 2.3. Measurement Instruments and Apparatus

The following anthropometric measurements were used: height (cm), weight (kg), and skinfolds (subscapular, medial calf, and triceps in mm). A portable Harpenden-stadiometer (Holtain, Ltd., Crymych, UK) was used to determine height to the closest 0.1 cm. Body weight was measured with an electronic scale to the nearest 0.1 kg (BF 511, Omron). Anatomical landmarks were drawn on the body to determine the subscapular, calf, and triceps skinfold measurements. Skinfold measurements were taken with a Harpenden skinfold caliper to the nearest 0.1 mm. Skinfolds were measured two to three times and the average value was used to ensure validity and reliability. These measurements were taken according to the ISAK protocol by post-graduate students in Human Movement Science specializing in Kinderkinetics that were all certified Level 3 Kinantropometrists and were executed according to the ISAK protocol (International Society for the Advancement of Kinanthropometry) [30]. Cutoff points compiled by Lohman (1992) [31] were used to calculate the sum of two skinfolds (triceps and subscapular). These two skinfolds display the strongest relation with body fat in children [32]. Fat percentage and lean body mass were also determined with a body composition monitor, OMRON, BF 511. Biological development was determined according to Tanner’s description of the five stages of breast development [33]. Breast development was assessed ranging from stage one to stage five where stage 1 represents no development and stage 5 the mature phase of this particular gender characteristic. All these measurements and assessments were conducted in private.

BMI was calculated by determining the relation between stature and weight (body weight in kg divided by length in m^2^). This study used international gender specific z-scores to classify girls as underweight (UW) or normal weight. The severity of thinness was determined by BMI percentiles in terms of grades of thinness (grade 1–3) where the following BMI classifications specified, grade 1 < 18.5 (a moderate presence), grade 2 < 17 (average presence) and grade 3 < 16 (serious presence). International BMI cutoff points (2007) were used as a set for age and gender [4].

Weight-for-age z-scores (WAZ) and height-for-age z-scores (HAZ) were also determined by using the WHO’s (1995) cutoff points to determine being underweight and stunting [32]. The results for z-scores with reference to WAZ are not available for girls older than 10 in the WHO Anthro software program (2005), as this indicator of body composition does not differentiate between height and body weight. A decrease in WAZ in the short-term is reflected in changes occurring in mass for height. At the age of 10, some girls experience a potential growth spurt due to puberty (WHO, Anthro: 2005) [32], which explains why WAZ could only be used for the older ages in this study.

#### Academic Performance

In grade 1 (2010), the assessment procedure of “The Mastery of Basic Learning Areas” was used to determine the degree to which the participants mastered basic academic literacy skills. Numeracy skills (mathematics), as well as reading and writing skills (handwriting standards and pencil grip) were assessed as prescribed by the DBE and assessed in South Africa. These skills were assessed according to four grading categories, namely: (1) no mastery; (2) partial mastery; (3) mastery; (4) outstanding mastery. These assessments were completed by the class teacher in June after the participants had spent five months in grade 1. These categories were converted to percentages for comparative purposes because the academic marks of grades 4 and 7′s were only reported as percentages.

The grade 4 and grade 7 academic progress reports in June 2013 and 2016 were obtained from the schools. These reports are divided into six learning areas according to the DBE’s Curriculum and Assessment Principle Declarations (CAPS) consisting of mathematics, home language, second language, natural sciences, social sciences, technology, and life orientation as well as the grade point average for all learning areas.

The results of the Annual National Assessment Tests (ANA), which include language and mathematics assessments that are annually written nationally in September, was made available by the DBE. These were used as a more objective academic performance mark. The ANA was replaced with the NWPA (North West Provincial Assessment) in 2016 because the ANA was not written in 2016 due to various reasons. The NWPA assessed academic performance in languages (Afrikaans, English, and Setswana) and mathematics on a provincial level.

### 2.4. Statistical Analysis

The Statistica computer program version 13.2 (StatSoft, Tulsa, OK, USA) [34] was used to analyze the data. The Anthro software program (2005) SAS version 9.4 (SAS Institute, Carry, NC, USA) was used to calculate the z-scores (WHO, Anthro: 2005) [31]. Data were descriptively analyzed as percentages, minimum and maximum values, and standard deviations. Frequency tables were compiled to determine the prevalence of normal weight, underweight, thinness, and stunting in every age group. The data were first checked for normality. By checking the distributions of the variables, no serious deviations of normality were detected. Independent *t*-testing was then used to determine time point differences with a statistical significance set at *p* < 0.05. A repeated measures ANOVA with a post hoc Bonferonni adjustment was used to determine statistically significant relations over time between underweight, thinness, stunting and academic performance. A *p*-value of < 0.05 was set as a statistically significant difference. The non-parametric Freedman test (F) was also used as verification if numbers shrank over time. All analyses were adjusted for school quintile status to correct for influences of different school environments.

## 3. Results

The descriptive anthropometric characteristics of the group, including height, weight, BMI, fat percentage, WAZ, HAZ, and BAZ (BMI-for-age z-score) are presented in Table 1. Higher academic performances are seen in grade 1 compared to in grades 4 and 7. The June school results revealed a significant decrease in mathematics from grade 1 (76.90%) to grade 7 (51.26%), with a similar decreasing trend in the grade point average (74.45% to 59.21%) over the six-year follow-up period.

Table 2 displays the body composition and academic performance of the participants as divided into four categories, namely stunting (S), thinness (T), underweight (UW), and normal weight at baseline and grade 4 and grade 7. It is important to note that the normal weight group was used as a reference group for comparison between the malnutrition categories, hence the normal weight group did not exclude girls that might have been overweight or obese. In grade 1, 20.2% (n = 40) of the group were stunted (S), 20.7% (n = 41) fell into the thinness (T) group while 21.7% (n = 43) were underweight (UW). In the Thinness group, 16.7% (n = 33) were classified with grade 1 thinness, 4.0% (n = 8) with grade 2 thinness, while no participants fell in the grade 3 thinness group. Changes in the number of participants in all three malnutrition groups are observed over the longitudinal period as participants moved in and out of the malnutrition groups during the follow-up period of six years. As described in the method, WAZ (weight–for age) z-scores could not be calculated for girls older than 10 years due to anthropometric and biological body changes in girls’ growing bodies after they turn ten.

The normal weight group was taller and heavier with higher mean anthropometric values compared to all the undernutrition categories, while also displaying better academic school performance than the S- and T-groups over the follow-up period. However, the UW-group performed better in all the academic measurements in grade 1 compared to the T- and S-groups. In grade 7, very similar mean values were achieved in the language provincial marks of the normal weight and the T-group and mathematics between the UW- and normal weight groups. Significant academic performance differences were found in grade 1 between the normal weight and S-group (mathematics *p* = 0.002); language *p* = 0.012; GPA (*p* = 0.004). In grade 4, performance in language in the provincial assessment revealed significant differences (*p* = 0.003) between these groups while significant differences appeared again in all academic school assessments (mathematics *p* = 0.028; language *p* = 0.030; GPA, *p* = 0.004) between the normal weight and S-group with the S-group presenting with poorer academic performance.

Table 3 portrays the time points differences and the results of a repeated measures ANOVA of academic differences between each of the undernutrition groups and the normal weight group over the follow-up period. The number of participants in the different groups in the table changed as a result of some participants following different pathways of nutrition status during the follow-up period and consequently moved between groups over the follow-up period.

Significant group differences were only found between the stunted and normal weight group in all academic measures at each time point and over time, where the S-group performed significantly poorer throughout (F = 10.43; *p* = 0.002).

The longitudinal group analysis over time* confirmed statistically significant differences in language skills (*p* = 0.007), mathematics (*p* = 0.001) and the GPA (*p* = 0.002). The S-group underperformed by almost 9% over the long-term compared to the normal weight group.

The academic results of the UW group were conflicting as they displayed higher mean values than the normal weight group in grade 1 (language = 75.00% opposed to 71.09%; mathematics = 78.57% opposed to 75.63%; GPA = 76.19% opposed to 72.62%). In grade 4 the opposite trend was observed where the normal weight group displayed higher percentages in language (58.77% opposed to 56.31%) and GPA (60.36% opposed to 59.16%) with similar mathematics percentages (UW group = 60.86%; normal weight group = 60.98%). In grade 7, the UW group performed better in language skills (64.69% opposed to 62.73%) and mathematics (49.48% opposed to 48.53%), with corresponding GPA values (UW = 57.80% and normal = 57.87%). None of these percentage differences between the underweight and normal weight groups were, however, significant at any time point or over time.

The thinness group underperformed academically compared to the normal weight group in almost all the time point academic comparisons in 2010, 2013, and 2016, although differences only reached significance in grade 1 in die GPA (*p* = 0.045). In grade 4, language marks differ up to 10% between the two groups (T = 49.12%; normal weight = 59.48%: *p* > 0.05). In grade 7, the mathematics performance of both groups display minor differences (T = 50.14%; normal = 50.71%). No significant differences were recorded over time between the T- and UW-groups.

**Figure 2:**
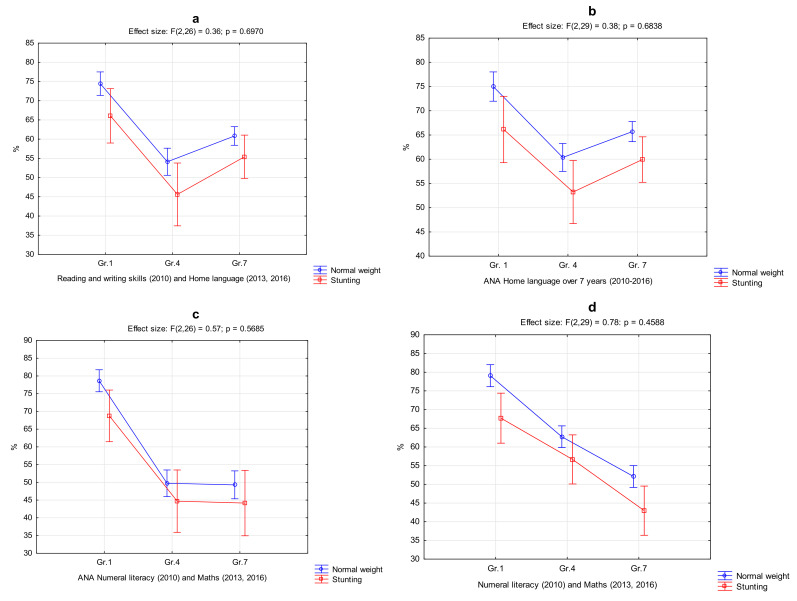

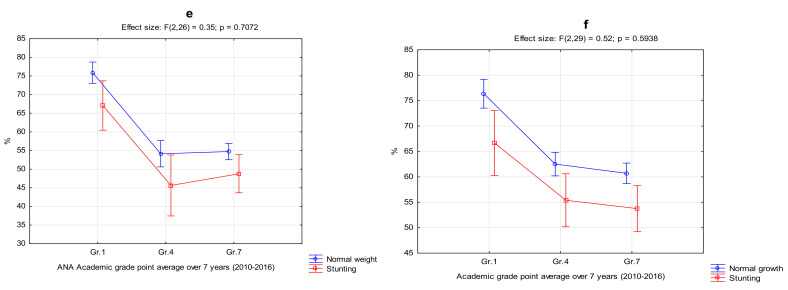
(**a**–**f**). Differences in academic performance between the school (2010—The Mastery of Basic Learning Areas; 2013 & 2016—June assessments) and provincial assessment (2010—ANA; 2013 & 2016—NWPA) of the normal weight (n = 123) and stunted BMI group (n = 26) during three follow-up time measurements.

These results confirm that only stunting had a negative and persistent impact on academic performance. The time point differences over time effect are subsequently illustrated graphically in Figure 2a–f only in language (home language and second language), mathematics, and the GPA between the stunted (n = 10; square) and the normal weight group (circle). Significant differences (*p* = 0.05) in academic performance between the two groups are portrayed in Table 3 and are visible from the graphs at all time point measures (2010–2016). No interaction effects (*p* > 0.05) were found between the groups. Although similar patterns of change in academic performance are visible from the graphs, it is clear that the academic performance path of both groups stayed the same from grade 1 to grade 7 where the normal weight group achieved significantly higher percentages in all academic comparisons over all time point comparisons. A widening of differences in language, mathematics, and GPA are also visible, especially in the grade 7 comparisons (Figure 2b,d,f).

## 4. Discussion

This study aimed to determine short term and long-term influences of stunting, thinness, and being underweight on academic performance over the primary school period of seven school years in girls. All three conditions showed a notable prevalence of at least 20 percent over the follow-up period. Over the longitudinal period, 17.4% (n = 26) of the group was stunted. This prevalence is lower than the reported 40% in Africa [3], although it still affected nearly one in five girls during the primary school years. Stunting also showed a tendency to affect more girls at older ages, especially over the period from six to nine years. In agreement, a study done in Kenya [26] reports that stunting is the most common form of undernutrition in school going children aged six to 12 years with a prevalence of 29.1% reported among girls.

The key finding confirmed that stunting had negative and lasting influences on academic performance in language, mathematics, and the grade point averages of girls based on the significantly poorer academic achievements of this group in grades 1, 4, and 7 as well as over the follow-up period of six years (F = 10.43; *p* = 0.002). Kar et al. [35] report in this regard that poor and insufficient nutrition during early childhood has direct influences on brain development, which is vital for higher cognitive processes. It is also reported that when inadequate nutrition persists over a long period, it may lead to permanent damage that will restrict cognitive development [35]. These results, therefore, confirm that stunting over the long-term can lead to the impediment of cognitive development by causing structural damage to the brain, as reported by Brown and Pollit [11]. Benton [14] confirms in this regard that undernutrition, which already occurs in the first year of life, shows long-term effects on cognitive abilities and behavior related aspects that lead to a lower intelligence quotient and poorer academic performance. Chesire et al. [26] also reported a statistically significant relation between school attendance and stunting. Although the current study did not analyze the specific link between stunting and school attendance, research worldwide confirms that the relation between stunting and school attendance could result in poor academic performance [9,20,21]. The WHO (2016) furthermore reports that poor school attendance due to stunting during childhood combined with poor educational outcomes can contribute to a decline of 22% in the potential annual income of an adult [36,37]. Stunting, therefore, seems to be a risk factor for survival and health during childhood and adulthood that can affect both learning capacity and productivity [3,21].

In agreement with our findings, various studies have also confirmed the negative consequences of different indices of undernutrition, for instance, underperformance in school [9,12,38]. The Young Lives longitudinal study over seven years in four developing countries representing similar populations to South Africa reported positive links between HAZ, language and mathematics, and overall scholastic performance among 8 to 15-year-olds [4]. Glewwe et al. [9] report findings from Philippine children that were followed from birth until the end of the primary school years where undernourished children reached school readiness at a much later stage and had to be held back more opposed to well-nourished children during their childhood. Similar findings are furthermore also reported by South African researchers based on cross-sectional studies [5,8]. A recent study on 8.3-year-old children conducted in four peri-urban quintile 3 public schools in the Port Elizabeth area of SA confirmed an association between stunting and poorer end of the year results among girls, although not among boys [8]. Pienaar [5] also report similar results as HAZ was positively associated with school performance. These researchers report a direct link between stunting and academic performance.

However, contradictory findings are also reported by a study in Malaysia [39] among 7–8-year-olds where no relation between poor nutrition (HAZ, WAZ, BAZ) and academic performance was reported. This study was, however, cross-sectional in nature and deemed other factors like SES, health-related conditions, and ethnicity as more important, which makes it difficult to compare the results directly with ours [39].

Although trends of lower academic performance were evident among thin and underweight girls, no statistically significant differences were found in their academic performance at each point or over time compared with normal weight girls (*p* ≤ 0.05, Table 3). In agreement, Haile et al. [40] also reported no links between WAZ and BAZ in overall academic performance and mathematics among children aged 8–11. This trend of lower academic performance, which was evident from our findings, could therefore rather be attributed to emotional responses to stressful circumstances caused by undernutrition [41] than to the impact of poor cognitive function and behavior. In addition, growing up in low SES circumstances is often accompanied by food insecurity and the largest percentage of the girls in the study originated from low socio-economic circumstances. Undernutrition can lead to children losing weight and having poor resistance to infections which, in turn, could become chronic in the long-term. If the latter transpires, it will not only affect school attendance, but also attention span and concentration [3,11].

The study has strengths due to the longitudinal nature of the analyses as well as the different indices of undernutrition that were analyzed. However, the study also had limitations that could have influenced the results. The large dropout rate of 200 girls over the follow-up period (50.3%) from 2010 to 2016 can affect the generalization of these findings. Only academic performance was used in this study, and not standardized tests which analyze other forms of mathematics and language abilities or intelligence quotients. The advantage of using academic marks is, however, that it brings general academic performance knowledge to the fore as well as indirect information on class attendance and behavior when the grade point average is used. Furthermore, the reference group which is referred to as the normal weight category did not exclude girls that might have been overweight or obese during this time.

Taking these weaknesses into account, the findings are still of importance because the sample of primary school girls could represent South Africa’s population composition. A further strength is that the participants were randomly selected and underwent longitudinal follow-up measurements that covered the whole primary school period of seven years. No similar studies, comparing South African girls’ academic performance and body composition over a follow-up period of six years, have been conducted in South Africa.

## 5. Conclusions

This study adds new information that confirms the persisting negative influence of stunting on the academic performance of primary school girls. This result confirms the importance of optimal nutrition for academic performance, as well as the significance of addressing inadequacies in this regard in order to create opportunities for a better quality of life for girls. Such support for young developing girls is vital to provide the next generation of South African mothers with a positive future as this research substantiates the prolonged negative consequences of undernutrition on girls. The outcome of this research is therefore of particular importance to plan and implement strategies to improve the health and subsequent wellbeing of girls. The significant relation that was established between academic underperformance and stunting among girls urge for the development and timely implementation of prevention strategies. Modifiable factors should be identified, especially by future studies, while government commitment is needed from various departments to address these developmental challenges in young children.

Empowering people such as mothers or mothers-to-be, daycare mothers, and teachers with knowledge and skills, especially in the lower SES environments, which are considered to be the breeding ground of malnutrition, is particularly important. Learning agricultural skills, but more importantly, the sustainable application of these skills at rural schools in cooperation with community-based feeding interventions, must be further pursued to ensure a practical and sustainable approach to food production at schools in the interest of the learners. Follow-up strategies should be implemented to determine the successes and shortcomings of these interventions. Doctors, in cooperation with the DBE, must also take responsibility to support and educate schools and parents, especially expectant mothers, daycare mothers, and preschool teachers about the importance of optimal nutrition for cognitive outcomes. It is recommended that similar studies must be conducted on adolescent girls as well as preschool girls to determine the effect of undernutrition on younger as well as older girls, together with similar studies in other South African provinces to strengthen the generalization of the results.

## Figures and Tables

**Table 1:** Descriptive characteristics of average body composition and academic performance at baseline and follow-up time points (*N* = 198).

	M ± SD	Min	Max
Stature (cm)			
*Gr.1*	119.06 ± 6.03	107.90	134.70
*Gr.4*	135.85 ± 7.13	113.00	155.10
*Gr.7*	153.85 ± 7.28	136.60	185.90
Body Mass (kg)			
*Gr.1*	22.20 ± 5.39	15.50	68.60
*Gr.4*	32.53 ± 9.98	20.50	113.10
*Gr.7*	47.77 ± 14.06	28.80	154.00
BMI			
*Gr.1*	15.54 ± 2.60	12.50	38.40
*Gr.4*	17.55 ± 4.64	12.26	61.24
*Gr.7*	20.01 ± 4.93	13.90	57.50
Body Fat (%)			
*Gr.1*	15.47 ± 4.99	7.59	31.81
*Gr.4*	20.92 ± 9.05	5.40	59.10
*Gr.7*	24.58 ± 8.29	10.10	47.60
∑Skinfolds(mm)			
*Gr.1*	16.60 ± 6.70	8.25	55.25
*Gr.4*	20.60 ± 10.54	7.50	72.00
*Gr.7*	24.71 ± 13.35	9.00	101.50
Z-Scores			
Gr.1			
*WAZ*	−0.12 ± 1.22	−2.71	7.73
*HAZ*	−0.13 ± 1.04	−2.65	2.70
*BAZ*	−0.09 ± 1.21	−2.25	7.54
Gr.4			
*WAZ*	−0.13 ± 1.39	−2.63	7.61
*HAZ*	−0.30 ± 1.05	−3.89	2.70
*BAZ*	0.07 ± 1.46	−5.15	8.81
Gr.7			
*WAZ*	-	-	-
*HAZ*	−0.26 ± 1.03	−2.78	4.29
*BAZ*	0.16 ± 1.45	−2.61	9.85
Academic performance (%)			
Gr.1			
*Language*	73.22 ± 17.45	25.00	100.00
*Mathematics*	76.90 ± 17.66	25.00	100.00
*GPA*	74.45 ± 16.52	25.00	100.00
Gr.4			
*Language*	59.60 ± 16.06	12.00	93.00
*Mathematics*	61.44 ± 16.21	25.00	95.00
*GPA*	61.43 ± 13.27	25.17	92.80
Gr.7			
*Language*	64.03 ± 11.52	18.00	91.50
*Mathematics*	51.26 ± 16.09	12.00	97.00
*GPA*	59.21 ± 11.07	20.33	92.56

BAZ = BMI-for-age z score; BMI = Body Mass Index; Body Fat % = fat percentage; GPA = grade point average; HAZ = height-for-age z score, M = mean, Max = maximum value; Min = minimum value; SD = standard deviation, ∑ Skinfolds = sum of supsc + triceps skinfolds, WAZ = weight-for-age z score; - = z-score omitted due to biological changes in girls older than 10.

**Table 2:** Body composition and academic performance differences between normal weight and girls with different forms of undernutrition in Gr.1, Gr. 4, and Gr. 7 (*N* = 198).

	Grade 1			Grade 4			Grade 7		
	**S**	**T**	**UW**	**S**	**T**	**UW**	**S**	**T**	**UW**
*Undernutrition Cat*	**n = 40**	**n = 41**	**n = 43**	**n = 48**	**n = 24**	**n = 34**	**n = 43**	**n = 33**	**n = 42**
	**M ±**	**M ±**	**M ±**	**M ±**	**M ±**	**M ±**	**M ±**	**M ±**	**M ±**
	**SD**	**SD**	**SD**	**SD**	**SD**	**SD**	**SD**	**SD**	**SD**
Age									
*Normal weight*	6.82 ±	6.78 * ±	6.83 ±	9.86 ±	9.81 * ±	9.8 ±	12.86 ±	12.87 ±	12.86 ±
	0.39	0.36	0.38	0.39	0.35	0.39	0.38	0.38	0.38
*Undernutrition Cat*	6.81 ±	6.96 * ±	6.78 ±	9.81 ±	10.14 * ±	9.81 ±	12.86 ±	12.81 ±	12.85 ±
	0.33	0.39	0.36	0.33	0.42	0.34	0.33	0.34	0.33
Stature (cm)									
*Normal weight*	120.88 * ±	119.17 ±	119.49 * ±	138.50 * ±	135.78 ±	136.33 * ±	156.30 * ±	154.63 * ±	154.47 * ±
	5.24	5.96	6.03	5.86	7.03	7.14	5.41	7.25	6.92
*Undernutrition Cat*	111.7 * 6 ±	118.47 ±	117.41 * ±	127.55 * ±	136.17 ±	133.46 * ±	144.89 * ±	149.81 * ±	151.43 * ±
	2.19	6.22	5.76	3.41	8.10	6.81	3.564	6.11	8.13
Body Mass (kg)									
*Normal weight*	23.08 * ±	22.45 ±	23.22 * ±	34.54* ±	32.63 ±	34.19 * ±	49.36 * ±	50.58 * ±	51.18 * ±
	5.57	5.79	5.57	10.21	10.21	10.18	11.44	13.99	14.11
*Undernutrition Cat*	18.59 * ±	21.36 ±	18.41 * ±	26.27 * ±	31.75 ±	24.54 * ±	42.61 * ±	34.47 * ±	35.69 * ±
	2.05	3.51	1.77	5.49	8.56	2.54	20.61	3.16	4.27
BMI (kg/m^2^)									
*Normal weight*	15.70 ±	15.67 ±	16.14 * ±	17.99 * ±	17.63 ±	18.28 * ±	20.14 ±	21.02 * ±	21.26 * ±
	2.78	2.80	2.61	4.87	4.76	4.77	3.98	4.96	4.98
*Undernutrition Cat*	14.88 ±	15.14 ±	13.34 * ±	16.19 * ±	16.97 ±	14.05 * ±	19.83 ±	15.33 * ±	15.65 * ±
	1.49	1.61	0.37	3.50	3.74	1.14	7.72	0.64	1.04
Body Fat (%)									
*Normal weight*	15.76 ±	15.46 ±	16.53 * ±	21.97 * ±	21.13 ±	22.88 * ±	25.14 ±	26.66 * ±	27.62 * ±
	5.21	5.26	4.94	9.98	8.89	8.37	7.94	778	7.78
*Undernutrition Cat*	14.19 ±	15.76 ±	11.52 * ±	17.55 * ±	19.43 ±	10.25 * ±	23.14 ±	15.02 * ±	15.94 * ±
	4.03	4.05	2.46	8.53	10.39	3.79	9.87	2.84	3.19
∑Skinfolds (mm)									
(*∑Supsc + Triceps*)									
*Normal weight*	17.01 ±	16.71 ±	17.85 * ±	22.05 * ±	20.86 ±	22.35 * ±	25.03 ±	26.93 * ±	27.62 * ±
	7.13	7.08	6.89	10.87	10.54	10.77	11.47	13.54	13.85
*Undernutrition Cat*	14.79 ±	16.54 ±	11.88 * ±	16.09 * ±	18.71 ±	12.20 * ±	24.27 ±	13.63 * ±	14.63 * ±
	4.03	5.06	2.46	8.15	10.80	2.54	19.19	2.33	2.99
School Assessment Maths									
*Normal weight*	78.82 * ±	78.04 ±	75.46 ±	62.36 ±	61.79 ±	61.52 ±	52.52 * ±	51.64 ±	50.28 ±
	16.30	18.26	19.44	16.02	16.60	16.35	16.18	16.70	21.47
*Undernutrition Cat*	69.38 * ±	72.56 ±	78.49 ±	58.62 ±	59.00 ±	61.18 ±	46.38 * ±	49.00 ±	50.41 ±
	20.79	14.58	18.57	16.82	13.35	15.97	15.14	12.72	18.71
Language									
*Normal weight*	74.92 * ±	74.12 ±	71.89 ±	60.34 ±	59.66 ±	59.35 ±	64.98 * ±	64.16 ±	64.06 ±
	16.84	17.68	16.96	16.63	16.30	16.08	10.87	11.68	11.69
*Undernutrition Cat*	67.19 * ±	70.42 ±	75.00 ±	57.14 ±	56.75 ±	51.39 ±	60.63 * ±	63.53 ±	64.04 ±
	18.28	16.00	18.11	14.14	15.80	11.06	13.29	10.87	11.07
Grade Point Av.									
*Normal weight*	76.22 * ±	75.43 ±	74.08 ±	62.25 ±	61.21 ±	61.69 ±	60.34 * ±	59.63 ±	53.59 ±
	15.64	16.82	17.04	13.60	13.80	13.57	10.77	11.40	12.35
*Undernutrition Cat*	67.9 * 2 ±	71.14 ±	76.16 ±	58.77 ±	62.90 ±	60.03 ±	54.85 * ±	56.79 ±	51.05 ±
	18.15	14.80	18.06	12.04	8.71	11.97	11.22	8.97	10.08
Provincial Marks (ANA/NWPA) Maths									
*Normal weight*	-	-	-	62.36 ±	61.79 * ±	61.72 ±	51.26 ±	50.42 ±	50.28 ±
				16.02	16.60	16.32	21.11	14.21	21.47
*Undernutrition Cat*	-	-	-	58.62 ±	59.94 * ±	56.561 ±	46.80 ±	49.71 ±	50.41 ±
				16.82	11.45	14.18	19.86	16.70	18.71
*Language*									
*Normal weight*	-	-	-	60.56 * ±	56.93 ±	58.28 ±	60.26 ±	59.22 ±	59.15 ±
				19.11	19.64	20.24	12.63	13.16	13.04
*Undernutrition Cat*	-	-	-	49.95 * ±	65.09 ±	56.59 ±	55.85 ±	59.85 ±	59.98 ±
				20.91	30.24	19.23	74.36	13.24	12.98

BMI = Body Mass Index; Body Fat % = fat percentage; Cat = category of undernutrition; M = mean; Subsc = subscapular skinfold; ∑ Skinfolds = sum of supsc + triceps skinfolds; SD = standard deviation; statistical significance = *p* < 0.05 *; S = stunting; T = thinness; UW = underweight; SD = standard deviation. Note: Gender specific cut-off points by Cole et al. were used for classification of thinness.

**Table 3:** Analysis of academic differences between groups per time point and over time.

**Stunting** **S (n = 26); Nw (n = 123)**										
	**2010**			**2013**			**2016**			**2010–2016**
	**S**	**Nw**		**S**	**Nw**		**S**	**Nw**		
	**M ±**	**M ±**	***p***	**M ±**	**M ±**	*p*	**M ±**	**M ±**	***p***	***p***
	**SD**	**SD**		**SD**	**SD**		**SD**	**SD**		
Language	66.83 ±	75.00 ±	0.026 *	53.30 ±	60.50±	0.043 *	54.42 ±	65.78 ±	0.017 *	0.007 *
	18.35	16.56		13.92	16.44		14.83	10.87		
Maths	68.27 ±	79.07 ±	0.003 *	57.80 ±	62.87 ±	0.160	43.48 ±	52.30 ±	0.015 *	0.001 *
	19.44	15.82		17.15	16.22		16.66	16.29		
GPA	67.31 ±	76.36 ±	0.009 *	56.13 ±	62.61 ±	0.024 *	60.72 ±	65.59 ±	0.004 *	0.002 *
	17.78	15.26		11.01	13.32		12.37	11.00		
**Underweight** **UW (n = 21); Nw (n = 112)**										
	**2010**			**2013**			**2016**			**2010–2016**
	**UW**	**Nw**		**UW**	**Nw**		**UW**	**Nw**		
	**M ±**	**M ±**	***p***	**M ±**	**M ±**	***p***	**M ±**	**M ±**	***p***	***p***
	**SD**	**SD**		**SD**	**SD**		**SD**	**SD**		
Language	75.00 ±	71.09 ±	0.380	56.31 ±	58.77 ±	0.506	64.69 ±	62.73 ±	0.484	>0.05
	18.11	18.76		15.44	15.52		10.70	11.90		
Maths	78.57 ±	75.67 ±	0.521	60.86 ±	60.98 ±	0.973	49.48 ±	48.53 ±	0.797	>0.05
	18.18	19.12		17.16	15.45		14.47	15.59		
GPA	76.19 ±	72.62 ±	0.395	59.16 ±	60.36 ±	0.695	57.80 ±	57.87 ±	0.977	>0.05
	17.34	17.67		10.85	13.30		9.40	11.13		
**Thinness** **T (n = 7); Nw (n = 147)**										
	**2010**			**2013**			**2016**			**2010–2016**
	**T**	**Nw**		**T**	**Nw**		**T**	**Nw**		
	**M ±**	**M ±**	***p***	**M ±**	**M ±**	***p***	**M ±**	**M ±**	***p***	***p***
	**SD**	**SD**		**SD**	**SD**		**SD**	**SD**		
Language	58.93 ±	72.60 ±	0.052	49.12 ±	59.48 ±	0.221	60.07 ±	63.77 ±	0.421	>0.05
	17.25	18.06		21.77	16.40		10.18	11.89		
Maths	64.29 ±	76.71 ±	0.078	60.12 ±	61.03 ±	0.156	50.14 ±	50.71 ±	0.929	>0.05
	19.67	18.02		22.02	15.80		18.60	16.17		
GPA	60.71 ±	73.97 ±	0.045 *	48.21±	52.47 ±	0.669	56.61 ±	59.17 ±	0.559	>0.05
	17.16	16.95		20.05	18.66		11.21	11.31		

M = mean; Nw = normal weight; SD = standard deviation; S = stunting; statistical significance * = *p* < 0.05; T = thinness; UW = underweight; GPA = grade point average.
